# Effect of magnesium sulphate on bi-spectral index (BIS) values during general anesthesia in children

**DOI:** 10.1186/s12871-015-0108-7

**Published:** 2015-09-22

**Authors:** Mahmoud Mostafa Amer, Ahmed Abdelaal Ahmed Mahmoud, Marwa Khaled Abdelrahman Mohammed, Ahmed Mostafa Elsharawy, Doaa Abo-elkasem Ahmed, Ehab Mohamed Farag

**Affiliations:** 1 Department of Anesthesia, Faculty of Medicine, Beni Suef University, Beni Suef, Egypt; 2 Department of Anesthesiology, Beni Suef University, 39 Mousa Ebn Nousir Street, 7th District, Nasr City, P.O. 11471, Cairo, Egypt

## Abstract

**Background:**

Magnesium was reported to reduce both the anesthetic requirements and the period needed to reach a bi-spectral index value of 60 when used intra-operatively (Br J Anaesth 83:302-20, 1999; Anesth Analg 20:1273-5, 1988; Br J Anaesth 89:594-8, 2002; Anesth Analg 87:206-10, 1998; Br J Anaesth 89:594-8, 2002; Br J Anaesth 94:438-41, 2005) and to minimize the emergence agitation (Anaesthesia 61:1058-63, 2006). Previous studies examined the influence of magnesium on the anesthetic requirements while the bi-spectral Index values were kept within a constant range. We evaluated the effect of intraoperative magnesium on the bi-spectral index values during pediatric anesthesia while we kept other anesthetic variables unchanged.

**Methods:**

Eighty pediatric patients with ASA physical status I, age 2–8 years and scheduled for minor infra-umbilical elective procedures included in a prospective randomized controlled study. We randomly divided patients into two groups. Group I (40 patients); received a bolus dose 50 mg/kg of magnesium sulphate followed by an infusion at rate of 15 mg/kg/h throughout the procedure. Group II (40 patients); received the same amount in the form of ringer acetate for blinding. We compared between the groups regarding: 1) BIS values. 2) Hemodynamic parameters. 3) Arterial oxygen saturation 4) End-tidal CO2 5) Respiratory rate and 6) Tidal volume.

**Results:**

Magnesium group (Group I) showed significantly lower BIS values and shorter time to achieve BIS values below 60. Respiratory parameters (tidal volume and respiratory rate) were significantly lower in the magnesium group. Otherwise, no significant differences between the study group and the control group were detected.

**Discussion:**

Our study has the advantage of evaluating the direct effect of magnesium sulphate on the Bi-spectral index scale with keeping other intraoperative factors almost constant (as the type of operations, induction and maintenance techniques, end-tidal anesthetic concentration, analgesia and mode of ventilation) for accurate assessment.

**Conclusion:**

Magnesium produced significantly lower BIS values, less time to reach BIS values below 60, lower tidal volume and lower respiratory rate during pediatric general anesthesia.

**Trial registration:**

Pan African Clinical Trial Registry, www.pactr.org, PACTR201312000666231. Registered at 6 October 2013.

## Background

Magnesium is a natural cation that represents the fourth most predominant cation in the body and the second most abundant intracellular cation. It has many essential biological functions as energy metabolism and nucleic acid synthesis, trans-membrane ion exchange and regulation of adenylate cyclase; muscle contractility; neuronal function and neuro-transmitter release. Magnesium has been known to be a physiological calcium antagonist [1].

Rats treated with magnesium showed a 60 % reduction in minimum alveolar concentration of halothane [2] this result was explained as a central effect of magnesium. Magnesium administration during total intravenous anaesthesia produced a significant reduction in the requirements for anaesthetic drugs; propofol, remifentanil and vecuronium [3].

Various studies [4–9] revealed that the perioperative magnesium can reduce the anaesthetic demands and the time needed to reach a BIS value of 60. In addition, magnesium has been reported to have anti-nociceptive effects in animal and human models of pain, and to reduce intraoperative analgesic consumption [4–9]. Moreover, magnesium could decrease post-operative agitation following tonsillectomy using sevoflurane anesthesia [10].

Although Bi-spectral index may not be accepted by some authors [11] as a monitor for depth of anesthesia superior to other methods yet BIS is the most accepted monitor of depth of sedation and anaesthesia in pediatric [12–17].

Previous studies [4–9] examined the effects of magnesium on the anesthetic doses needed to keep bi-spectral Index values within a fixed range (40–60).

Our present randomized controlled study evaluated the influence of intraoperative magnesium on BIS values while keeping other anesthetic variables almost constant during pediatric anesthesia.

## Methods

We carried out a prospective, randomized controlled study at Beni-Suef University Hospital in the period from the May 2013, to September 2013, after approval by the local research and ethics committee (Faculty of medicine- Beni Suef University- Research Ethical Committee). The trial was registered at Pan African Clinical Trial Registry (trial number: PACTR201312000666231).

**Fig. 1:**
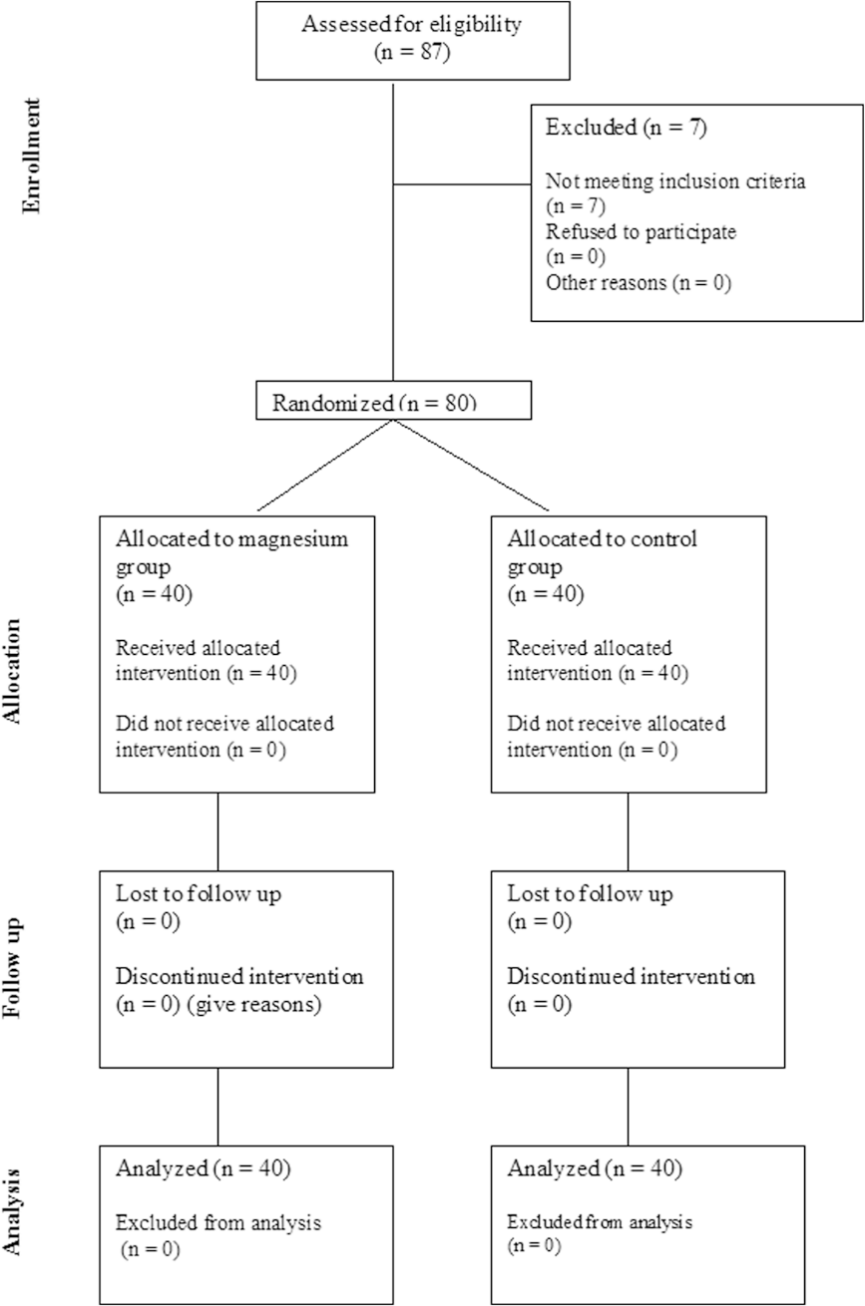
CONSORT flowchart showing the number of patients at each phase of the study

We obtained a written form of informed consent from the parents in charge. We included 80 children (Fig. 1), ASA physical status I, aged 2–8 years scheduled for minor infra-umbilical surgeries in the supine position (e.g. herniorrhaphy, hypospadias, hydrocele etc.) in the study. Exclusion criteria included patients with head anomalies (e.g. microcephaly, hydrocephalus), Down syndrome, cerebral palsy, brain tumors, epilepsy and patients scheduled for major or head and neck surgery or surgeries in non-supine position. Patients underwent routine preoperative evaluation including history, examination and investigations.

According to the protocol in our hospital, patients received premedication with intramuscular midazolam 0.05 mg/ kg and atropine 0.01 mg/kg thirty minutes before induction of anesthesia by inhalation of sevoflurane aiming for end-tidal sevoflurane concentration of 2 volume % through a face mask. An intravenous cannula was inserted after loss of consciousness. For randomization, a computer-generated random numbers was used and the numbers were concealed in opaque sealed envelopes. Patients were randomly assigned to either group I (Magnesium) or group II (Control). Group I (*n* = 40) received magnesium sulphate with a priming dose of 50 mg/kg over ten minutes. Infusion started immediately after insertion of an intravenous line followed by an infusion at rate of 15 mg/kg/h throughout the procedure. Group II (*n* = 40) received balanced solution (ringer acetate) with the same volume and infusion rate as for group I with the attended anesthetist was unaware which solution was used for blinding. After the application of standard monitoring, an inhalation of sevoflurane ensured an adequate depth of anesthesia with end-tidal sevoflurane concentration of 2 volume %.

The patient’s airway was secured by laryngeal mask airway (LMA™) with the appropriate size used according to the weight of the patient. The volume of cuff inflation was according to the recommendations of the manufacturer. The LMA™ was connected to a standard pediatric circle circuit connected to the anesthesia workstation (Zeus® Drӓger anaesthesia workstation, Drӓger Medical, Lübeck, Germany) with side stream capnogram. Manual ventilation was performed to ensure proper position of LMA™ by obtaining three square-shaped waves of capnography with adequate chest expansion. Manual assisted ventilated until spontaneous respiration resumed was done. The patients continued with unassisted spontaneous ventilation.

For intraoperative analgesia, all patients received caudal epidural block (bupivacaine 0.25 % at a volume of 1 ml/kg) and paracetamol suppository at a dose of 15 mg/kg. End- tidal sevoflurane concentration was maintained at 2 volume %. The inspired sevoflurane concentration was not recorded as it was changing rapidly and controlled automatically together with the fresh gas flow by the anesthesia machine (Zeus® Drӓger anaesthesia workstation, Drӓger Medical, Lübeck, Germany) in order to maintain expired sevoflurane concentration of 2 volume %.

Respiratory parameters that included respiratory rate, expired tidal volume, end-tidal CO2, and SpO2 were monitored continuously and recorded every ten minutes throughout the procedure. Tidal volume was measured using a spirometer sensor at the patient’s end. Side stream capnography was used to monitor end-tidal carbon dioxide concentration. The electrodes of the bi-spectral index were applied on the patient’s forehead, and BIS values were recorded immediately after induction of anesthesia then every ten minutes and till end of the operation. Hemodynamic parameters that included heart rate, systolic blood pressure and diastolic blood pressure were monitored continuously and recorded every ten minutes. The mean values of each of these parameters were computed for each patient, and these values were used for statistical analysis.

At the end of the surgery, we stopped the infusion of a balanced solution or magnesium sulphate, discontinued inhaled anesthetic, removed LMA while the patient was at sevoflurane end tidal concentration of 2 volume %. Then we allowed the patient to emerge from anesthesia then transferred to PACU. We recorded any complications while conducting anesthesia, recovery or at PACU.

### Statistical analysis

Assuming α error = 0.05 (two-tailed) and β error = 0.1. Sample size of 34 patients, allocated into one group, will have a power of 90 % to determine an assumed clinically significant difference of 5 % (effect size d = 0.6) or more between the paired measurements of BIS value in magnesium and control groups. *t*- Test for matched pairs was used to estimate the sample size. The sample size was calculated using Power Analysis and Sample Size 12 software (NCSS, Kaysville, UT, USA).

Data are expressed as mean ± SD. Analysis of variance (ANOVA) test was used for comparison between means of the two groups. For all comparisons, we considered *P* < 0.05 statistically significant. We used software version 21 of statistical package for social science (SPSS version 21, Chicago, IL, USA).

## Results

The study included 80 patients in two equal groups; Group I (magnesium group), Group II (control group). We did not detect statistically significant differences between the groups regarding age, sex, weight and performed surgical procedures (Table 1).

**Table 1:** Demographic data and surgical procedures in both groups

	Group I (Magnesium)	Group II (Control)	*P* value
Age (years)	5.7 ± 2.1	6.4 ± 2.2	0.731
Sex (M/F)	17/23	21/19	0.124
Weight (kg)	20.3 ± 4.5	22.4 ± 5.6	0.694
Surgical procedures			
Inguinal herniorrhaphy	17	25	0.08
Hypospadias	14	10	0.07
Hydrocele	6	4	0.09
Undescended testis	2	1	0.06
Cystoscopy	1	0	0.06

*M* male, *F* female, *Kg* kilogram

Data of age and weight are as mean ± standard deviation

*P* values ≤ 0.05 are considered statistically significant

No significant difference between the groups was reported regarding hemodynamic parameters (heart rate, systolic blood pressure and diastolic blood pressure) (Table 2).

**Table 2:** Hemodynamic parameters in both groups

	HR			SBP			DBP		
	Group I	Group II	*P* value	Group I	Group II	*P* value	Group I	Group II	*P* value
After induction	112.9 ± 15.1	118.8 ± 16.3	0.630	102.4 ± 10.1	108.9 ± 11.9	0.281	61.6 ± 8.7	65.7 ± 6.6	0.374
10 min.	109.9 ± 13.3	113.6 ± 15.5	0.746	103.5 ± 10	107.6 ± 10.8	0.317	64.2 ± 10.3	64.8 ± 6.3	0.709
20 min.	106 ± 13.3	109 ± 14.8	0.821	102 ± 10.5	106.7 ± 11.4	0.477	65.5 ± 9.8	64.2 ± 6.5	0.532
30 min.	103 ± 12.4	104.3 ± 14.6	0.955	100.5 ± 10.5	105.7 ± 10.7	0.395	63.1 ± 10	64.4 ± 6	0.803
40 min.	99.2 ± 13	102.4 ± 13.1	0.852	96.7 ± 9.1	103.7 ± 11.5	0.271	61.4 ± 9	63.2 ± 7.4	0.857
50 min.	93.4 ± 11.9	99.3 ± 12.6	0.483	97.9 ± 9.9	99.6 ± 9.5	0.728	61.9 ± 9.5	61.6 ± 7.3	0.987
60 min.	89.1 ± 11.2	96.2 ± 11.7	0.411	102.7 ± 14.2	98.3 ± 7.9	0.677	61 ± 9.6	61.9 ± 7.6	0.596
70 min.	89.7 ± 10.5	91.5 ± 17.7	0.990	103.3 ± 12.1	102.5 ± 10.6	0.673	60 ± 16.5	68 ± 9.9	0.803
80 min.	87 ± 8.9	89.5 ± 17.7	0.978	102 ± 6.9	102 ± 14.1	0.732	59.3 ± 13.7	66.5 ± 12	0.786

Group I (Magnesium sulphate group)

Group II (Control group)

Data are as mean ± standard deviation

*SBP* systolic blood pressure

*DBP* diastolic blood pressure

*HR* heart rate

*P* values ≤ 0.05 are considered statistically significant

BIS values were lower in the magnesium group than in the control group, and this reduction was statistically significant at 20 to 80 min of intraoperative time (Table 3). All patients in the magnesium group reached BIS values less than 60 in 20 min while patients in the control group required 30 min to do so, this depended on the highest BIS value within the group and not the mean BIS value for the group.

**Table 3:** Bispectral index scale (BIS) values in both groups

Bispectral index scale (BIS) values	Group I (mean ± SD)	Group II (mean ± SD)	*P*-value
After induction	66.8 ± 6.5	67.1 ± 5.2	0.894
10 min.	61.7 ± 5.4	65.6 ± 4.8	0.055
20 min.	56.1 ± 3.2	63.7 ± 2.3	0.041*
30 min.	52.6 ± 5.4	56.2 ± 3.6	0.036*
40 min.	52.3 ± 4.5	55.3 ± 2.4	0.045*
50 min.	48.1 ± 4.7	55.2 ± 4.6	0.040*
60 min.	45.9 ± 3.4	53.2 ± 4.9	0.047*
70 min.	45.2 ± 1.3	52.6 ± 6.1	0.043*
80 min.	46.3 ± 1.5	51.1 ± 5.6	0.041*

Group I: magnesium group, Group II: control group

Data are as mean ± SD

*Statistically significant difference

*BIS* Bispectral Index Scale

Tidal volume was lower in the magnesium group than in the control group, and this decrease was statistically significant throughout the operation (Table 4).

**Table 4:** Tidal volume (ml) in both groups

Tidal volume	Group I (Magnesium) mean ± SD	Group II (Control) mean ± SD	*P*-value
After induction	131.4 ± 43.9	176.3 ± 44.5	0.003*
10 min.	131.0 ± 40.8	177.8 ± 42.5	0.001*
20 min.	131.5 ± 38.4	175.5 ± 43.8	0.002*
30 min.	129.8 ± 42.3	173.4 ± 44.2	0.003*
40 min.	129.6 ± 38.8	170.9 ± 38.4	0.002*
50 min.	128.9 ± 39.5	169.5 ± 38.1	0.002*
60 min.	125.9 ± 36.8	169.3 ± 38.3	0.001*
70 min.	123.0 ± 42.1	170.0 ± 39.9	0.013*
80 min.	133.9 ± 43.8	185.0 ± 30.4	0.006*

Group I: magnesium group, Group II: control group

Data are as mean ± SD

*Statistically significant

*ml* milliliter

Respiratory rate was significantly lower in the magnesium group till 60 min of the intra-operative time (Table 5).

**Table 5:** Respiratory rate in both groups

Respiratory rate	Group I (Magnesium) mean ± SD	Group II (Control) mean ± SD	*P*-value
After induction	23.8 ± 0.6	30.2 ± 1.3	0.001*
10 min.	23.8 ± 0.6	28.3 ± 1.3	0.001*
20 min.	24.2 ± 0.7	26.3 ± 1.0	0.002*
30 min.	23.3 ± 0.7	25.4 ± 1.0	0.002*
40 min.	22.4 ± 0.5	25.2 ± 1.3	0.012*
50 min.	22.5 ± 0.8	25.1 ± 1.3	0.054*
60 min.	22.5 ± 0.8	25.2 ± 1.3	0.034*
70 min.	24.5 ± 0.9	25.1 ± 1.5	0.280
80 min.	24.7 ± 1.0	24.8 ± 1.4	0.897

Group I: magnesium group, Group II: control group

Data are as mean ± SD

*Statistically significant

Respiratory rate is measured in a breath per minute

We did not detect statistically significant difference in End-tidal carbon dioxide concentration (Table 6) or arterial oxygen saturation.

**Table 6:** End tidal carbon dioxide (mmHg) in both groups

End tidal CO_2_	Group I (Magnesium) mean ± SD	Group II (Control) mean ± SD	*P*-value
After induction	39.6 ± 13.9	37.3 ± 1.6	0.466
10 min.	39.7 ± 15.5	37.3 ± 1.5	0.486
20 min.	40.2 ± 17.7	36.8 ± 1.4	0.397
30 min.	39.7 ± 16.6	36.4 ± 1.4	0.375
40 min.	39.1 ± 14.2	36.0 ± 1.4	0.330
50 min.	38.9 ± 13.3	35.8 ± 1.4	0.299
60 min.	38.9 ± 14.3	35.5 ± 1.6	0.303
70 min.	36.6 ± 1.7	35.3 ± 1.6	0.650
80 min.	37.1 ± 1.3	34.5 ± 1.6	0.522

Group I: magnesium group, Group II: control group

Data are as mean ± SD

*mmHg* millimeter mercury

In conclusion, magnesium sulphate group had lower BIS values, shorter time to achieve BIS values below 60, lower tidal volume, and lower respiratory rate with no significant differences in hemodynamic parameters or end-tidal CO2 were reported. In either group, no complications were reported.

## Discussion

Our study revealed that magnesium sulphate significantly reduced the time needed to achieve a BIS value below 60 for all patients (20 min. in the magnesium group versus 30 min. in the control group). Magnesium significantly reduced the BIS values, tidal volume and the respiratory rate. The end-tidal CO2 was comparable between the groups. No significant difference in hemodynamic parameters and no complications were reported.

Although there is no solid evidence for the use of BIS as a method to prevent awareness in pediatric anesthesia, there is no doubt that BIS is a useful monitor for depth of anesthesia. We used BIS to evaluate the central effect of magnesium as BIS has been used in pediatric age group as an adequate monitor for level of sedation and anesthesia [12–17]. Also BIS was considered as a valuable method to prevent awareness in the practice of anesthesia with an adequate level of general anesthesia and a low risk of intra-operative awareness accompany BIS values of 40 to 60 [18].

Our study has the advantage of evaluating the direct effect of magnesium sulphate on the Bi-spectral index scale with keeping other intraoperative factors almost constant (as the type of operations, induction and maintenance techniques, end-tidal anesthetic concentration, analgesia and mode of ventilation) for accurate assessment. While previous studies [4–9] that examined the effect of using intra-operative magnesium depended on evaluating the change in the demanded doses of intravenous anesthetics, inhaled anesthetics, narcotics or muscle relaxants to keep BIS values within a constant range, that BIS range was from 40 to 60 which may be considered a relatively wide range to be relied on to evaluate the effect of magnesium on anesthetic requirements. In other words, it is easier to control drug doses and measure variation in BIS values to evaluate the direct effect of magnesium rather than keeping BIS values within a relatively wide range (40–60) and measuring drug dosing. Also drug dosing is an indirect measure of the central effect of magnesium while BIS values can be considered as a direct monitor of that effect.

Our study is one of the few studies [10, 19–21] that examined the effects of intraoperative magnesium sulphate in pediatric anesthesia. These studies [10, 19–21] examined the effect of magnesium on intraoperative muscle relaxants requirement [21] or post-operative emergence agitation [10, 19, 20]. But they did not examine the effect of magnesium on bi-spectral index.

Magnesium sulphate has been used intravenously at dose of 25–75 mg/kg [22] for treatment of asthma in children in five randomized trials [23–27]. For magnesium dosing, we followed H.S. Na et al. [21] who used magnesium at dose of 50 mg/kg bolus followed by 15 mg/kg without reporting any side effects.

Lim B. et al. [28] concluded that pressure support ventilation did not produce significant clinical outcome when compared with spontaneous ventilation during pediatric anesthesia increased tidal volume and decreased end-tidal carbon dioxide. We used spontaneous ventilation in our study to examine the effect of magnesium sulphate on respiratory parameters.

Our study revealed that the magnesium produced a significant decrease in respiratory rate and tidal volume without affecting end-tidal CO2 concentration or oxygen saturation that can be explained by anesthetic and analgesic properties of magnesium. No hemodynamic instability or adverse events were reported.

Although no hemodynamic side effects were reported in our study, it is important to note that we used atropine as a premedication in all recruited patients according to the local protocol in our hospital. This may alter the accuracy of estimating possible hemodynamic effects of magnesium in an age group characterized by heart rate dependent cardiac output.

In conclusion, our study represents another evidence for the effect of magnesium sulphate as an adjunct to general anesthesia, but this time in the pediatric age group that has received a limited number of studies. We also used the bi-spectral index scale as a monitor to assess the direct central effect of magnesium sulphate.

### Limitations and future plan

One of the limitations in our study is that we did not measure perioperative plasma concentration of magnesium. Correlation between magnesium plasma level and BIS values can be useful in a future study for more accurate assessment of the direct central effect of magnesium sulphate guided by the BIS.

## Conclusion

Magnesium sulphate as an adjuvant to general anesthesia in pediatric patients remarkably reduced time to reach BIS values of 60 by about 10 min and produced significantly less BIS values. Magnesium resulted in significantly lower tidal volume, lower respiratory rate but without a statistically significant hypercapnia. We did not report any hemodynamic instability or complications.

## Abbreviations

BIS: Bispectral index scale
Co2: Carbon dioxide
ASA: American society of anesthesiology
FMBSU-ERC: Faculty of medicine at Beni Suef University-Ethical and research committee
LMA: Laryngeal mask airway
CPAP: Continuous positive airway pressure
PACU: Post anesthesia care unit
*P*-value: Probability value
SD: Standard deviation

## References

[1] Fawcett WJ, Haxby EJ, Male DA (1999). Magnesium: physiology and pharmacology. Br J Anaesth.

[2] Thompson SW, Moscicki JC, Difazio CA (1988). The anesthetic contribution of magnesium sulphate and ritodrine hydro chloride in rats. Anesth Analg.

[3] Telci L, Esen F, Akcora D, Erden T, Canbolat AT, Akpir K (2002). Evaluation of effects of magnesium sulphate in reducing intraoperative anaesthetic requirements. Br J Anaesth.

[4] Koinig H, Wallner T, Marhofer P, Andel H, Hörauf K, Mayer N (1998). Magnesium sulphate reduces intra and postoperative analgesic requirements. Anesth Analg.

[5] Ray M, Bhattacharjee DP, Hajra B, Pal R, Chatterjee N. Effect of clonidine and magnesium sulphate on anaesthetic consumption, haemodynamics and postoperative recovery: A comparative study. Indian J Anaesth. 2010;54(2):137–41. 10.4103/0019-5049.63659 PMC2900737 20661352

[6] Altan A, Turgut N, Yildiz F, Türkmen A, Ustün H (2005). Effects of magnesium sulphate and clonidine on propofol consumption, haemodynamic and post-operative recovery. Br J Anaesth.

[7] Gupta K, Vohra V, Sood J (2006). The role of magnesium as an adjuvant during general anesthesia. Anaesthesia.

[8] Lee DH, Kwon IC (2009). Magnesium sulphate has beneficial effects as an adjuvant during general anesthesia for caesarean section. Br J Anaesth.

[9] Khafagy HF, Ebied RS, Osman ES, Ali MZ, Samhan YM (2012). Perioperative effects of various anesthetic adjuvants with TIVA guided by bispectral index. Korean J Anesthesiol.

[10] Abdulatif M, Ahmed A, Mukhtar A, Badawy S (2013). The effect of magnesium sulphate infusion on the incidence and severity of emergence agitation in children undergoing adenotonsillectomy using sevoflurane anaesthesia. Anaesthesia.

[11] Dag C, Bezgin T, Özalp N, Gölcüklü AG (2014). Utility of bispectral index monitoring during deep sedation in pediatric dental patients. J Clin Pediatr Dent.

[12] Sadhasivam S, Ganesh A, Robison A, Kaye R, Watcha MF (2006). Validation of the bispectral index monitor for measuring the depth of sedation in children. Anesth Analg.

[13] Orliaguet GA, Lambert FB, Chazot T, Glasman P, Fischler M, Liu N. Feasibility of closed-loop titration of propofol and remifentanil guided by the bispectral monitor in pediatric and adolescent patients: a prospective randomized study. Anesthesiology. 2014;122(4):759–67. 10.1097/ALN.0000000000000577 25545655

[14] McDermott NB, VanSickle T, Motas D, Friesen RH (2003). Validation of the bispectral index monitor during conscious and deep sedation in children. Anesth Analg.

[15] Shields CH, Styadi-Park G, McCown MY, Creamer KM (2005). Clinical utility of the bispectral index score when compared to the University of Michigan Sedation Scale in assessing the depth of outpatient pediatric sedation. Clin Pediatr (Phila).

[16] Powers KS, Nazarian EB, Tapyrik SA, Kohli SM, Yin H, van der Jagt EW (2005). Bispectral index as a guide for titration of propofol during procedural sedation among children. Pediatrics.

[17] Malviya S, Voepel-Lewis T, Tait AR, Watcha MF, Sadhasivam S, Friesen RH (2007). Effect of age and sedative agent on the accuracy of bispectral index in detecting depth of sedation in children. Pediatrics.

[18] Avidan MS, Zhang L, Burnside BA, Finkel KJ, Searleman AC, Jacqueline A (2008). Anesthesia awareness and the bispectral index. N Engl J Med.

[19] Bondok RS, Ali RM (2014). Magnesium sulfate reduces sevoflurane-induced emergence agitation in pediatric patients. Ain-Shams J Anaesthesiol.

[20] Abu-Sinna RG, Talat SM (2011). The effect of a pre-induction bolus dose of magnesium sulphate on emergence agitation after sevoflurane anesthesia in children undergoing adenotonsillectomy. Ain shams J Anaesthesiol.

[21] Na HS, Lee JH, Hwang JY, Ryu JH, Han SH, Jeon YT (2010). Effects of magnesium sulphate on intraoperative neuromuscular blocking agent requirements and postoperative analgesia in children with cerebral palsy. Br J Anaesth.

[22] Bichara MD, Goldman RD (2009). Magnesium for treatment of asthma in children. Can Fam Physician.

[23] Scarfone RJ, Loiselle JM, Joffe MD, Mull CC, Stiller S, Thompson K (2000). A randomized trial of magnesium in the emergency department treatment of children with asthma. Ann Emerg Med.

[24] Ciarallo L, Sauer AH, Shannon MW (1996). Intravenous magnesium therapy for moderate to severe pediatric asthma: results of a randomized, placebo-controlled trial. J Pediatr.

[25] Devi PR, Kumar L, Singhi SC, Prasad R, Singh M (1997). Intravenous magnesium sulfate in acute severe asthma not responding to conventional therapy. Indian Pediatr.

[26] Gürkan F, Haspolat K, Bosnak M, Dikici B, Derman O, Ece A (1999). Intravenous magnesium sulphate in the management of moderate to severe acute asthmatic children nonresponding to conventional therapy. Eur J Emerg Med.

[27] Ciarallo L, Brousseau D, Reinert S (2000). Higher-dose intravenous magnesium therapy for children with moderate to severe acute asthma. Arch Pediatr Adolesc Med.

[28] Lim B, Pawar D, Ng O (2012). Pressure support ventilation vs. spontaneous ventilation via ProSeal™ laryngeal mask airway in pediatric patients undergoing ambulatory surgery: a randomized controlled trial. Paediatr Anaesth.

